# A composite symptoms severity score based on survey self-reports as a predictor of SARS-CoV-2 infection and viral load

**DOI:** 10.1186/s12879-025-11653-4

**Published:** 2025-09-23

**Authors:** Damian Diaz, Jesse A. Canchola, Ana M. Groh, Tuna Toptan, Daniel Jarem, Alison L. Kuchta, Priscilla Moonsamy, Annemarie Berger, Maria J. G. T. Vehreschild, Sandra Ciesek

**Affiliations:** 1 Department of Internal Medicine, Infectious Diseases, Goethe University Frankfurt, University Hospital Frankfurt, Frankfurt am Main, Germany; 2 Roche Molecular Systems, Pleasanton, CA USA; 3 Institute of Medical Virology, Goethe University Frankfurt, University Hospital Frankfurt, Frankfurt am Main, Germany; 4 Fraunhofer Institute for Translational Medicine and Pharmacology ITMP, Frankfurt am Main, Germany; 5 Present address: Institute for Virology, TUM School of Medicine and Health, Munich, Germany

**Keywords:** SARS-CoV-2, Symptom severity, Viral load, Viral transmission

## Abstract

**Background:**

Establishing a strong correlation between active SARS-CoV-2 infection and COVID-19 severity could enhance early risk assessment, predict disease outcomes, and identify patients needing urgent treatment.

**Methods:**

In this prospective SARS-CoV-2 transmission cohort study, we introduce the potential of a symptoms severity score (S3) based on patient self-reported symptoms and further evaluate its utility for predicting SARS-CoV-2 infection status and viral load. The S3 construct, derived from a participant survey using pre-defined scales (Cronbach’s alpha=0.7), was categorized as asymptomatic, mild to moderate, or severe. This analysis comprised nine household contacts, contributing 1,410 qualitative and 89 quantitative visit‑test observations.

**Results:**

S3 showed a high correlation with total symptoms (Pearson *r* = 0.963, *p* < 0.0001). The categorized version (S3C) also correlated strongly with the number of symptoms (Spearman’s *r* = 0.988, *p* < 0.0001). A generalized estimating equation (GEE) model revealed that participants with severe symptoms had 6.5 times higher odds of having an active SARS-CoV-2 infection than those with no symptoms (Odds Ratio = 6.5, 95% CI: 3.5 to 12.4, *p* < 0.0001). Similar significant results were found for severe vs. mild to moderate symptoms (OR = 2.3, CI: 1.3 to 4.1, *p* = 0.0025) and mild to moderate vs. asymptomatic (OR = 2.8, 95% CI: 1.4 to 5.4, *p* = 0.0030).

**Conclusions:**

Our findings demonstrate that self-reported symptom severity and number of symptoms are robust predictors of SARS-CoV-2 infection and viral load, providing potential utility in clinical risk stratification. However, limitations, including a small sample size for viral load analyses and reliance on self-reported data, should be considered.

**Supplementary Information:**

The online version contains supplementary material available at 10.1186/s12879-025-11653-4.

## Background

In this prospective cohort study of household SARS-CoV-2 transmission, we explored the relationship between a novel Symptoms Severity Score (S3), derived from patient-reported symptoms on pre-defined scales, and the number of symptoms reported by each patient. We evaluated both metrics as potential predictors of SARS-CoV-2 infection and viral load (VL), addressing the urgent need for early indicators of SARS-CoV-2 positivity and VL. Identifying correlations between disease presence and symptoms severity may enhance early risk assessment and prediction of COVID-19 outcomes [1–3]. Furthermore, it could support prioritizing treatment for those most at risk and contribute to reducing the spread of infection.

## Methods

A prospective cohort study of households with a SARS-CoV-2 positive index patient (IP) and SARS-CoV-2 negative household contacts (HHC) was conducted, which primarily evaluated viral transmission dynamics and biomarker profiles (Groh et al., 2024). IPs were recruited into the prospective cohort study at the time of their initial SARS-CoV-2 diagnosis, which was confirmed by reverse transcription-polymerase chain reaction (RT-PCR) at the acute care clinic at University Hospital Frankfurt. The subsequent testing mentioned in the manuscript refers to the planned, longitudinal follow-up testing protocol for the study. As part of the study design, both the IPs and their household contacts (HHCs) were tested regularly with RT-PCR over a 30-day period to monitor viral transmission dynamics and biomarker profiles. More specifically, in the Methods it is noted that IPs were recruited ≤ 48 h after diagnostic RT-PCR and tested daily on days 0–7, then every 3–4 days until day 30 ± 6. SARS-CoV-2 infection status of each IP and HHC was evaluated using two RT-PCR tests in nasal swab specimens. A computer-assisted self-interview (CASI) questionnaire was developed for this study (Additional Material. 1). An excel document was created from the developed questionnaire and at each prescribed visit, the questionnaire was given to participants to gather information about the type and severity of up to 12 symptoms (Table 1). Full study details have been previously described [4]. Ethical approvals were obtained from Ethikkommission des Fachbereichs Medizin der Goethe-Universität c/o Universitätsklinikum (Reference number: 2021-119-MPG) and all procedures were performed in accordance with the relevant guidelines and regulations, and each participant provided informed consent.

### Data points used for analyses

This analysis includes the nine HHCs who became positive for SARS-CoV-2 during the study period after suspected exposure to the infected IP in the participant’s household. Each HHC had between 7 and 13 visits. At each visit, these HHCs were tested for SARS-CoV-2 infection. For positive tests, the HHC were tested at that and all subsequent visits to determine viral level. Multiple test types and repeat testing were conducted at each visit (see section “Quantitative analyses of SARS-CoV-2”). Negative nucleic acid amplification tests (NAAT) results were imputed as zero for analysis purposes. This approach simplifies interpretation and assumes undetectable VL for negatives. Any visits without testing or symptoms data were excluded from further analysis.

The resulting number of data points for each analysis type are as follows: qualitative analyses, all non-antibody HHC Tests and Visits (*n* = 1410); quantitative analyses, E gene SARS-CoV-2 Tests and Visits (*n* = 89).

### Number of symptoms derivation

Each of the 12 possible patient symptoms was coded as indicator variables: “1” if the symptom was indicated and as a “0”, otherwise. The total number of symptoms (NoS) was derived as the sum of indicator variables for each of the 12 symptoms at each visit, which were further grouped into three categories (NoSC): 0 symptoms, 1–2 symptoms and 3 + symptoms. Note that a simpler (categorized) representation of the number of symptoms may still capture the essential statistical information contained in the continuous version. This aligns with the statistical principle of *sufficiency* (Casella & Berger, 2002), whereby a reduced statistic can retain the same—or nearly the same—inferential content about the parameter of interest as the full dataset.

### Symptoms severity score derivation

A symptoms severity score (S3) was derived from the patient self-reported symptoms following an approach to measure symptoms severity as follows. First, we assigned values to the reported symptoms based on their severity (Table 1). The severity values for eight symptoms (cough, breathing difficulty, fatigue, body aches (myalgia), headache, sore throat, congestion/runny nose, and nausea) were categorized as follows: “None"=0, “Mild"=1, “Moderate"=2, and “Severe"=3 based on patient responses. For vomit and diarrhoea, the assigned values were: “0"=0, “1–2"=1.5, and “3–4"=3.5 based on symptom frequency. For loss of taste and loss of smell, the assigned values were: “Same as usual"=0, “Less than usual"=1.5, and “No taste or smell"=3.5.

**Table 1:** Symptoms items and recodes in patient CASI questionnaire

Item	Possible Response Set	Numerical Recode
1. Cough	0 = None, 1 = Mild, 2 = Moderate, 3 = Severe	0=”None”, 1=”Mild”, 2=”Moderate”, 3=”Severe”
2. Shortness of Breath or Difficulty Breathing		
3. Fatigue		
4. Muscle or body aches		
5. Headache		
6. Sore Throat		
7. Congestion or runny nose		
8. Nausea		
9. Vomit (within the previous 24 h)	0 = None, 1–2 times*, 3–4 times*	0=”None”, 1.5=”1–2 times”, 3.5=”3–4 times”
10. Diarrhea (within the previous 24 h)		
11. My sense of taste is	* Same as usual, Less than usual, I have no sense of taste	0=”Same as usual”, 1.5=”Less than usual”, 3.5=”I have no sense of taste”
12. My sense of smell is	* Same as usual, Less than usual, I have no sense of smell	0=”Same as usual”, 1.5=”Less than usual”, 3.5=”I have no sense of smell”

*CASI*, computer-assisted self-interview

* Note that these items were asked as is and were not originally provided with a numeric value

A symptoms severity score (S3) was calculated by summing the recoded values across all visits for each subject to obtain a comprehensive measure of symptoms severity (see Supplementary Material S.1 and Additional Material 1).

For a simpler representation of symptoms severity, the S3 construct values were grouped into three categories (S3C): an S3 value of 0 indicated *asymptomatic* individuals, a score of 1–2 denoted *mildly to moderately symptomatic* individuals, and a score of 3 or higher represented *severely symptomatic* individuals. To note, these representations refer to actual survey response choices in this study (see Table 1), and should not be confused with the CDC [5] or WHO definitions [6].

### Quantitative analyses of SARS-CoV-2

Quantitative analyses were carried out using the Cobas^®^ SARS-CoV-2 assay for use on the Cobas 6800/8800 System (Roche Diagnostics). NAAT were conducted for E gene SARS-CoV-2 and ORF1 gene SARS-CoV-2. Of the two available cycle threshold (Ct) values reported, only the E gene SARS-CoV-2 Ct was used as the results were very similar between the two targets for all SARS-CoV-2 positive patient visits. The quantitation scheme used to transform this semi-quantitative result can be found in the Supplementary Materials section, S.2.

### Statistical analysis

#### S3 construct assessment

The internal consistency of the S3 construct was assessed using the standardized Cronbach’s alpha (Ca), a measure of reliability [7]. To confirm the underlying dimensionality of the reduced 12-item set, a factor analysis with varimax rotation was conducted [8–11].

#### Descriptive statistics

The Spearman rank correlation coefficient was used to assess the correlations between S3 with the Number of Symptoms (NoS). To assess the association between S3C and NoSC the Rao-Scott modified chi – squared test was used. Pearson product‑moment correlations were also calculated for normally distributed variables and are reported where appropriate. Given that S3 is mathematically derived from the same 12 symptom items counted in NoS, this head‑to‑head comparison illustrates near‑collinearity and supports use of the simpler NoS when an ordinal scale is unavailable.

In addition, the cross tabulation between S3C and NoS with qualitative VL result (i.e., positive/negative) was constructed. A Taylor linearization approach was used to account for multiple visits per participant [12], and stratification by various study testing assays was used.

Adjusted descriptive statistics, including the mean, median, standard deviation, minimum, and maximum values of log_10_ copies/mL E gene SARS-CoV-2 VL (see Supplementary Material), were calculated for results in each of the S3C and NoSC categories.

Additional normalizing transformations using the logarithm base 10 (log_10_) were performed as needed on S3 and NoS continuous-level variables (i.e., S3Log and NoSLog, respectively).

### Modelling

A generalized estimation equation (GEE) marginal model [13–16] accounting for the correlation of subjects within multiple visits within the same participant was performed to model the relationship between the following:

A. S3C and the binary test outcome (i.e., RNA positive/negative);
B. NoSC and the binary test outcome;
C. S3Log and the Log_10_ VL outcome;
D. NoSLog and the Log_10_ VL outcome;
E. S3 and the Log_10_ VL outcome;
F. NoS and the Log_10_ VL outcome (see Supplementary Material 3 for C–F).

The models for binary test outcomes employed a binomial distribution and logit link, adjusted for within-subject correlated visit data with a specified working correlation structure. The continuous outcome models with Log_10_ VL used a normal distribution and identity link and similarly adjusted for correlated data also with a so-called specified working correlation structure. To model the within-subject dependencies, we evaluated three working correlation structures: (a) compound symmetric (exchangeable), (b) AR [1] or autoregressive with lag 1, and (c) independent (SAS Institute, 2016). To find the optimal working correlation structure for each model, the *Q*uasi likelihood under the *I*ndependence model *C*riterion (QIC) goodness-of-fit (GOF) statistic [15, 17] was computed to assess the model fit, with a lower value indicating a better fit of the model with the specific correlation structure. Table 2 shows the GEE models considered.

**Table 2:** GEE models for SARS-CoV-2 outcomes (as qualitative and quantitative) with various symptoms realizations as model predictor. Includes acronym definitions

Model	SARS-CoV-2 Outcome	Symptoms Realization as Predictor
1	SARS-CoV-2 qualitative result (RNA positive/negative) across all visits and tests (*n* = 1410)	S3C: three category Symptoms Severity Score. “Asymptomatic”, “Mild symptoms”, “Severe symptoms”
2	SARS-CoV-2 qualitative result (RNA positive/negative) across all visits and tests (*n* = 1410)	NoSC: Number of Symptoms Category “0 symptoms”, “1–2 symptoms”, “3 + symptoms”
3A	Log10 VL: log_10_ cp/mL E gene SARS-CoV-2 VL (*n* = 89)	S3Log: Log_10_ of Symptoms Severity Score (S3) construct
4A	Log10 VL: log_10_ cp/mL E gene SARS-CoV-2 VL (*n* = 89)	NoSLog: Log_10_ of the number of symptoms (NoS) variable
3B	Log10 VL: log_10_ cp/mL E gene SARS-CoV-2 VL (*n* = 89)	S3: Symptoms Severity Score (S3) construct
4B	Log10 VL: log_10_ cp/mL E gene SARS-CoV-2 VL (*n* = 89)	NoS: Number of Symptoms (NoS) variable

*cp/mL* copies per milliliter, *VL* viral load

Models 1 and 2 are equivalent to logistic regression, but within the GEE framework, they account for data correlation induced by multiple visits per patient. These two GEE models are specified using a binomial distribution, logit Link function, and an assumed or working correlation structure for the data. Analogously, Models 3A, 3B, 4A, 4B correspond to linear regression and, under the GEE framework, also address data correlation. The latter two models employ a normal distribution and identity link function while assuming a working correlation structure to account for within-subject dependencies.

Estimates from the categorical analysis models are reported as odds ratios (OR) along with their 95% confidence interval. P-values less than 0.05 were considered statistically significant. All analyses were carried out using SAS v9.4 [12].

## Results

### Description of the S3 construct

The right-skewed distribution of S3 (Figure S.1) highlights the concentration of lower-severity symptom reports, reflecting real-world variability in symptoms severity among participants. The Ca value for the all-symptoms score was 0.70. The range of 0.70 to 0.79 suggests moderate internal consistency, indicating that the items in the scale construct are reasonably correlated with each other [18]. To confirm the underlying dimensionality of the reduced 12-item set, a factor analysis with varimax rotation was conducted. The results affirmed that the combined symptoms score represented a single underlying dimension defined here as the Symptoms Severity Score or S3.

### Correlation between symptoms severity score and number of symptoms

The Pearson correlation between S3 and total symptoms was 0.963 (*p* < 0.0001). For the E gene SARS-CoV-2 (SC2) target subsample (*n* = 89), the Pearson correlation between S3Log with the NoSLog was 0.9773 (*p* < 0.0001). For the categorical versions with all results (*n* = 1410), the Spearman rank correlation between S3C and NoSC was 0.9880 (*p* < 0.0001).

### Correlation between S3/S3Log and nos/noslog with Log_10_ VL

The Pearson correlation coefficient between S3Log and log_10_ VL was 0.28 (*p* = 0.0073) and 0.30 (*p* = 0.0046) with the untransformed S3. Similarly, the correlation between NoSLog and log_10_ VL was 0.27 (*p* = 0.0092) and 0.26 (*p* = 0.0141) with the untransformed NoS. A sensitivity analysis imputing log10 VL = 0 for qualitative negatives yielded similar but attenuated slopes (data not shown), confirming robustness to the testing algorithm.

### Association between S3/S3C and nos/nosc with viral positivity

The crosstabulation between (S3C and NoSC) with infection status is shown in Table 3. The Rao-Scott modified chi-square test, with 2 degrees of freedom, yielded statistically significant p-values (less than 0.0001) for the two underlying tables (S3C and NoSC) presented in Table 3. The positivity rate increased as symptoms severity increased from asymptomatic to mild/moderate symptoms to severe symptoms for S3C (12.5%, 28.6%, 48.3%, respectively) and from 0 symptoms to 1–2 symptoms to 3 or more symptoms for NoSC (12.5%, 27.5%, 55.7%, respectively).

**Table 3:** Association between S3C and NoSC with viral positivity

Categorical Symptoms Constructs (Rao-Scott modified chi-square with 2 df, p-value)*		SARS-CoV-2 Positivity (All tests, *n* = 1410)		
		% Positive		
		Estimate	95% LL**	95% UL**
S3C (38.7, < 0.0001)	Asymptomatic	12.5	7.3	17.7
	Mild to Moderate Symptoms	28.6	18.8	38.3
	Severe Symptoms	48.3	37.8	58.9
NoSC (45.9, < 0.0001)	0 Symptoms	12.5	7.3	17.7
	1–2 Symptoms	27.5	18.6	36.4
	3 + Symptoms	55.7	44.0	67.3

*LL *lower limit, *S3C* three category Symptoms Severity Score, *NoSC* Number of Symptoms Category, *UL* upper limit

* Reflects adjustment based on clustering of patients with multiple visits and stratification by various study testing assays

** Variance estimation based on Taylor linearization correcting for clustering of patients with multiple visits and stratification by various study testing assays

### Modelling

GEE linear models accounting for the correlation of subjects within multiple visits were performed to model the relationship between two SARS-CoV-2 outcome types (qualitative for Models 1 and 2 and quantitative for Models 3A, 3B, 4A, 4B) and four symptoms realizations as predictors (categorical for Models 1 and 2, respectively: S3C, NoSC, and continuous for Models 3A, 3B and Models 4A, 4B, respectively: S3Log/S3, NoSLog/NoS); see Table 2 for the modelling strategy). However, the potential for the relationship between symptom severity and VL to change over the course of infection is an important consideration. Initially, a GEE model was used which accounts for the correlation of repeated measurements within the same individual over the 30-day follow-up period. By assessing different working correlation structures, we aimed to statistically model the longitudinal nature of the data. It is noteworthy that this does not explicitly illustrate how the relationship between symptoms and VL evolves over time. Therefore, after evaluating the time by symptom interaction and a week-specific GEE, the output indicates that there is justification for statistical homogeneity across time, allowing the focus of the paper to be on the overall effect.

1. The omnibus-interaction tests were non-significant for both binary infection and log-VL outcomes (week by symptom χ²=12.3, degrees of freedom (df) = 10, *p* = 0.27; χ²=7.8, df = 5, *p* = 0.17, respectively).
2. The main-effect “week” terms are also non-significant (*p* ≈ 0.29–0.78), while the symptom main-effects remain robust.

For the categorical analyses using Models 1 and 2, the results showed the independent working correlation to be the best-fitting model using the QIC criterion as shown in Table 4. For continuous-level analyses using Models 3A, 3B, 4A and 4B, the results showed the compound symmetric (CS; also known as “exchangeable”) correlation structure for the proper modelling of the correlation relationship within the data. The lower QIC values for the independent structure in Models 1–2 suggest minimal within‑participant autocorrelation for binary infection status, whereas the compound symmetric fit in Models 3–4 indicates stable intra‑subject VL covariance across visits.

**Table 4:** GEE model correlation structure assessment for models 1 to 4B

Correlation Structure	QIC* Goodness-of-Fit Statistic					
	Categorical Analysis (*n* = 1410)		Continuous Analysis (*n* = 89)			
	Model 1	Model 2	Model 3A	Model 4A	Model 3B	Model 4B
CS	1444	1428	88	88	88	88
AR(1)	1455	1464	91	91	92	92
Independent	1377	1356	91	91	92	92
Equation						
Outcome	Pos/Neg SC2	Pos/Neg SC2	log_10_ cp/mL E gene SC2	log_10_ cp/mL E gene SC2	log_10_ cp/mL E gene SC2	log_10_ cp/mL E gene SC2
Predictor	S3C	NoSC	log_10_ S3 (S3Log)	log_10_ NoS (NoSLog)	S3	NoS

*AR* [1] auto regressive with lag 1, *CS* compound symmetric (same as exchangeable), *GEE* generalized estimating equation accounting for multiple visits within patient and stratification by assay test, *neg* negative, *NoSC* number of symptoms category, *pos* positive, *SC2* SARS-CoV-2 *S3C *three category Symptoms Severity Score

*Quasi likelihood under the Independence model Criterion goodness-of-fit statistic, Lower values indicate better-fitting models. Bolded QIC values represent the chosen correlation structure for that model. ** Correlation structure used for the specific model

### Model 1 for positivity as outcome with categorical predictor S3C

Table 5 shows the resulting GEE Model 1 results. The model used S3C as the predictor of SARS-CoV-2 infection status with results showing that those participants who reported severe symptoms in the survey had an odds 6.5 times higher to be classified as positive (vs. negative) than those who reported no symptoms (Odds Ratio = 6.5, 95% CI: 3.5 to 12.4, *p* < 0.0001). Similar statistically significant results were found when comparing *severe symptoms* vs. *mild to moderate symptoms* (OR = 2.3, CI: 1.3 to 4.1, *p* = 0.0025) and *mild to moderate symptoms* vs. *asymptomatic* reports (OR = 2.8, 95% CI: 1.4 to 5.4, *p* = 0.003).

**Table 5:** Odds ratio results of GEE model 1 (with independent correlation structure) for SARS-CoV-2 status (RNA positive/negative) outcome with symptoms severity category (S3C) as a predictor (*n* = 1410)

S3C	Statistic	Estimate	Standard Error	95% Confidence Interval		Chi-Square	*P*-value
Severe vs. Asymptomatic(ref)	Log Odds	1.88	0.33	1.24	2.52	33.22	< 0.0001
	Odds Ratio	6.54	2.13	3.46	12.39		
Mild to Moderate vs. Asymptomatic(ref)	Log Odds	1.03	0.34	0.36	1.69	9.14	0.0025
	Odds Ratio	2.80	0.95	1.44	5.45		
Severe vs. Mild to Moderate(ref)	Log Odds	0.85	0.29	0.29	1.41	8.83	0.0030
	Odds Ratio	2.34	0.67	1.34	4.10		

*Asymp* asymptomatic, *GEE* generalized estimating equation accounting for multiple visits within patient and stratification by assay test, *ref* reference level

### Model 2 for positivity as the outcome with categorical predictor NoSC

Table 6 shows the resulting GEE Model 2 results with the model using S3C as the predictor of SARS-CoV-2 infection status. Participants who reported 3 or more symptoms in the survey had almost 9 times higher odds of being classified as positive (vs. negative) than those who reported zero symptoms (OR = 8.8, 95% CI: 4.5 to 17.1, *p* < 0.0001). Similar statistically significant results were found when comparing 3 or more symptoms vs. 1 to 2 symptoms (OR = 3.3, 95% CI: 1.9 to 5.9, *p* < 0.000) and 1 to 2 symptoms vs. zero reports (OR = 2.7, CI: 1.4 to 5.1, *p* = 0.0035).

**Table 6:** Results of GEE model 2 (with independent correlation structure) for SARS-CoV-2 infection status outcome with number of symptoms category (NoSC) as a predictor (*n* = 1410)

S3C	Statistic	Estimate	Standard Error	95% Confidence Interval		Chi-Square	P-value
3 + Symptoms vs. Asymptomatic(ref)	Log Odds	2.17	0.34	1.51	2.84	40.95	< 0.0001
	Odds Ratio	8.78	2.98	4.51	17.07		
1–2 Symptoms vs. Asymptomatic(ref)	Log Odds	0.98	0.33	0.32	1.63	8.51	0.0035
	Odds Ratio	2.65	0.89	1.38	5.12		
3 + Symptoms vs. 1–2 Symptoms(ref)	Log Odds	1.20	0.29	0.62	1.77	16.44	< 0.0001
	Odds Ratio	3.31	0.98	1.85	5.89		

*GEE* generalized estimating equation accounting for multiple visits within patient and stratification by assay test, *ref* reference level

## Discussion

The results of this study demonstrate an association between self-reported symptoms severity, number of symptoms, SARS-CoV-2 infection status, and VL. Both the S3 and NoS were correlated with each other (both as continuous and categorical realizations), with qualitative viral test positivity, and with continuous VL outcomes. The qualitative analysis showed that participants reporting severe symptoms, or three or more symptoms, had substantially higher odds of being classified as testing positive for SARS-CoV-2 RNA compared to those who were asymptomatic [19, 20].

In scenarios where a severity score has not been documented, such as in an electronic medical record in a retrospective study, the number of symptoms can serve as a reasonable proxy. This is supported by the strong correlation observed between the number of symptoms and SARS-CoV-2 infection status. The data suggest that individuals reporting three or more symptoms are significantly more likely to test positive for viral RNA, making the number of symptoms a practical and reliable indicator in the absence of detailed severity scoring [21].

The VL analyses with a smaller data set further strengthened these findings. Both the continuous S3 and NoS variables, as well as their log-transformed versions, exhibited a positive monotonic relationship with log_10_ VL as shown in Figures S2–5. In other words, as symptoms severity and number of symptoms increased, so did the predicted VL level. This graded association relationship reinforces the link between symptomatology and the underlying viral burden driving COVID-19 severity [22].

Collectively, these results suggest that patient self-assessments of symptoms presence and intensity could serve as an accessible proxy for SARS-CoV-2 VL and disease severity when clinical testing is non-existent or unavailable. Simple questionnaires capturing key symptoms or symptom counts could play a vital role in triaging high-risk individuals, particularly in resource-constrained settings. These tools may complement laboratory testing to prioritize care for severe cases while enabling earlier isolation to reduce transmission.

### Limitations

While the results demonstrate clear associations between symptom measures and SARS-CoV-2 VL, this study has several important Limitations. Firstly, this study has a small sample size to determine quantitative outcomes. The quantitative VL analyses were restricted to the nine household contacts, yielding a total of 89 visit where a SARS-CoV-2 test was qualitatively positive. As a result, the “asymptomatic” group within the VL analysis (e.g., in Table S.1) consists of individuals who had a detectable VL, but did not report symptoms at that specific visit. This selection process necessarily excludes asymptomatic individuals who were test-negative (and thus had a true VL of 0), which introduces a selection bias and inflates the mean VL reported for the asymptomatic category. We addressed this fundamental difference in data structure by performing two distinct sets of analyses:

1. Qualitative analysis (Models 1 & 2): To assess the odds of testing positive, we used the full dataset of 1410 visits, which included all negative results. This provided an unbiased assessment of the association between symptoms and the likelihood of having a detectable infection.
2. Quantitative analysis (Models 3 & 4): To assess the relationship between symptoms and viral burden, we analyzed the subset of 89 positive visits, modeling the VL conditional on being infected.

All data were derived from a single prospective household cohort in Germany, reducing the statistical power and generalisability. Additional studies across different geographic settings are necessary to confirm the broader applicability of the associations between symptoms and VL. Additionally, VLs were quantified using an average calculated from previously published standard curves, rather than a formally validated reference. Also, the symptom severity score (S3) used in this study has not yet been validated in an external population. Consequently, VL accuracy and reproducibility could vary across different testing systems. Further research is needed to verify its reliability, validity, and relevance in broader clinical and research contexts. Finally, as symptom–VL dynamics are expected to change over time, week‑specific regressions incorporating a symptom-by-time interaction were performed. While effect estimates were directionally consistent across weeks, the reduced sample sizes within strata resulted in wider CIs.

As symptoms and associated severity scores were self-reported by patients, the data may be subject to reporting biases, potentially impacting the accuracy of the results. Additionally, during the course of the study, some participants received antiviral treatments which could have altered disease progression, reduced the time to a clinical recovery, and altered the symptom profiles [23], further impacting the results. Furthermore, the participant survey focused solely on COVID-19, meaning the data does not differentiate SARS-CoV-2 from other respiratory viruses such as influenza. Future analyses could benefit from clinical evaluations to reduce the self-reporting potential bias and improve reliability.

The study design required that participant symptoms were recorded at predetermined study visits rather than continuously, thereby limiting the ability to capture fluctuations in symptom severity that may occur between visits. More frequent or continuous monitoring could provide a more comprehensive understanding on symptom variability. Additionally, participant baseline factors such as vaccination history, previous infections, and underlying comorbidities were not addressed in this analysis. These factors may influence the relationships between symptom severity and viral load.

Finally, this study reflects SARS-CoV-2 variants circulating in Germany between June 2021 and October 2022 [4], which may no longer be the predominant strain [24]. Therefore, our findings may not be applicable to newer strains that could exhibit different symptom profiles or patterns of severity.

Despite these limitations, the strengths of this study include the prospective longitudinal design, the use of a symptoms scale constructs, and robust statistical modelling approaches that accounted for within-subject correlations. The converging evidence from multiple analyses consistently pointing to symptom-positivity and symptom-VL associations provides proof-of-concept but also warrants further investigation in larger cohorts with VL quantitation methods based on an accepted reference standard not available during the study period. In addition, future studies should investigate the potential of clustering symptoms into categories (e.g., respiratory, systemic) to refine the predictive utility of S3 and explore whether certain symptom types correlate more strongly with viral load.

## Conclusions

This study demonstrates a robust link between self-reported COVID-19 symptoms (both severity and number) and SARS-CoV-2 status and, to a limited extent, to viral load levels, suggesting potential clinical value in using symptom information to complement laboratory testing. These findings highlight the utility of self-reported symptoms in augmenting laboratory diagnostics to inform patient triage, treatment prioritization, and infection control strategies. Future research is needed to validate these results in larger, more diverse cohorts and adapt them to evolving variants.

## Supplementary Information

Supplementary Material 1.

Supplementary Material 2.

## Data Availability

The study was conducted in accordance with applicable regulations. For more information on the study and data sharing, qualified researchers may contact the corresponding author, Dr Annemarie Berger, at annemarie.berger@em.uni-frankfurt.de.

## Abbreviations

CASI: Computer-assisted self-interview
COVID-19: Coronavirus disease 19
Ct: Cycle threshold
GEE: Generalised estimating equation
GOF: Goodness-of-fit
HHC: Household contact
IP: Index patient
NAAT: Nucleic acid amplification test
NoS: Number of symptoms
QIC: Quasi likelihood under the independence model criterion
RT-PCR: Reverse transcription-polymerase chain reaction
S3: Symptom severity score
S3C: Symptom Severity score categorised
SARS-CoV-2: Severe acute respiratory syndrome coronavirus 2
VL: Viral load
