# Supplementary material for: A composite symptoms severity score based on survey self-reports as a predictor of SARS-CoV-2 infection and viral load
Source: BMC Infect Dis. 2025 Sep 23;25:1116. doi: 10.1186/s12879-025-11653-4 (PMC12455794; doi:10.1186/s12879-025-11653-4)
Supplement: Supplementary file 1 — Supplementary Material 1. [file 12879_2025_11653_MOESM1_ESM.pdf]

Site	Subject ID	Protocol
____________________	____________________	**RDS-BRAVA-541**

At Home Visit Questionnaire (IP)

**At Home Visit #**	**Date of Visit**
__________	__ __ /__ __ __ /__ __ __ __<br>DD MMM YYYY

1. Since the last visit, have you been in close contact with anyone **outside** of your household? ☐ Yes ☐ No
   a. ***If Yes**, what was the duration of contact:* ☐ *< 15min* ☐ *15min - 1 hr* ☐ *> 1 hr*
   b. ***If Yes**, were you wearing a mask?* ☐ *Yes* ☐ *No*
   c. ***If Yes**, did you wash or sanitize your hands after?* ☐ *Yes* ☐ *No*
2. Since the last visit, have you been in close contact with anyone **inside** of your household? ☐ Yes ☐ No
   a. ***If Yes**, what was the duration of contact?* ☐ *< 15min* ☐ *15min - 1 hr* ☐ *> 1 hr*
   b. ***If Yes**, were you wearing a mask?* ☐ *Yes* ☐ *No*
   c. ***If Yes**, did you wash or sanitize your hands after?* ☐ *Yes* ☐ *No*
3. Since the last visit, have you had any non-household visitors to your household? ☐ Yes ☐ No
   a. ***If Yes**, what was the duration of the visit?:* ☐ *< 15min* ☐ *15min - 1 hr* ☐ *> 1 hr*
   b. ***If Yes**, were you wearing a mask?* ☐ *Yes* ☐ *No*
   c. ***If Yes**, did you wash or sanitize your hands after?* ☐ *Yes* ☐ *No*
4. Since the last visit, have you had an unscheduled healthcare visit (e.g. Telehealth, Clinic Visit, emergency room, hospital admission, etc.)? ☐ Yes ☐ No
   a. ***If Yes***, describe:

Site	Subject ID	Protocol
___________________________	___________________________	**RDS-BRAVA-541** ___________________________

5. Have you started any new medications since the last visit?<br><br>a. ***If Yes***, describe:	☐ Yes ☐ No
6. Have you received a COVID-19 vaccination since the last visit?	☐ Yes ☐ No<br>If Yes:<br>Vaccine manufacturer:<br>__________________<br>Dates of injection:<br>Dose 1: ____________<br>Dose 2:____________ ☐ N/A
7. Has your living situation changed since the last visit (e.g. new housemate, housemate moved, etc.)?<br><br>a. ***If Yes***, describe:	☐ Yes ☐ No
8. Have you become pregnant since the last visit?	☐ Yes ☐ No ☐ N/A
9. Are you currently breastfeeding?	☐ Yes ☐ No ☐ N/A

Site	Subject ID	Protocol
______________________	______________________	**RDS-BRAVA-541** ______________________

At Home Visit Questionnaire (HHC)

**At Home Visit #**	**Date of Visit**
__________	__ __ /__ __ __ /__ __ __ __<br>DD MMM YYYY

1. Since the last visit, have you been in close contact with anyone **outside** of your household?	☐ Yes ☐ No
a. ***If Yes****, what was the duration of contact:*	☐ *< 15min* ☐ *15min - 1 hr* ☐ *> 1 hr*
b. ***If Yes****, were you wearing a mask?*	☐ *Yes* ☐ *No*
c. ***If Yes****, did you wash or sanitize your hands after?*	☐ *Yes* ☐ *No*
2. Since the last visit, have you been in close contact with anyone who has shown symptoms of COVID-19 or has tested positive for COVID-19 (including the index patient in this study)?	☐ Yes ☐ No
a. ***If Yes****, what was the duration of contact:*	☐ *< 15min* ☐ *15min - 1 hr* ☐ *> 1 hr*
b. ***If Yes****, were you wearing a mask?*	☐ *Yes* ☐ *No*
c. ***If Yes****, did you wash or sanitize your hands after?*	☐ *Yes* ☐ *No*
3. Have you had any non-household visitors to your household since the last visit?	☐ Yes ☐ No
a. ***If Yes****, what was the duration of contact:*	☐ *< 15min* ☐ *15min - 1 hr* ☐ *> 1 hr*
b. ***If Yes****, were you wearing a mask?*	☐ *Yes* ☐ *No*
c. ***If Yes****, did you wash or sanitize your hands after?*	☐ *Yes* ☐ *No*
4. Since the last visit, have you had an unscheduled healthcare visit (e.g. Telehealth, Clinic Visit, emergency room, hospital admission, etc.)?<br>a. ***If Yes***, describe:	☐ Yes ☐ No

Site	Subject ID	Protocol
___________________________	___________________________	**RDS-BRAVA-541**<br>___________________________

5. Have you started any new medications since the last visit?<br>a. ***If Yes***, describe:	☐ Yes ☐ No
6. Have you received a COVID vaccination since the last visit?	☐ Yes ☐ No<br>If Yes:<br>Vaccine manufacturer:<br>____________________<br>Dates of injection:<br>Dose 1: _____________<br>Dose 2:_____________ ☐ N/A
7. Has your living situation changed since the last visit (e.g. new housemate, housemate moved, etc.)?<br>a. ***If Yes***, describe:	☐ Yes ☐ No
8. Have you become pregnant since the last visit?	☐ Yes ☐ No ☐ N/A
9. Are you currently breastfeeding?	☐ Yes ☐ No ☐ N/A

Site	Subject ID	Protocol
___________________________	___________________________	**RDS-BRAVA-541** ___________________________

Subject Symptom Diary (IP/HHC)	Date and Time of form completion<br>__ __ /__ __ __ /__ __ __ __ __ __ :__ __<br>DD MMM YYYY HH MM	Temperature<br>________℃<br>☐ Not Done
Symptom	0 = None 1 = Mild 2 = Moderate 3 = Severe	In the last 24 hours, has the symptom:<br>1 = improved<br>2 = worsened<br>3 = stayed the same
Cough	☐ 0 ☐ 1 ☐ 2 ☐ 3	☐ 1 ☐ 2 ☐ 3
Shortness of Breath or Difficulty Breathing	☐ 0 ☐ 1 ☐ 2 ☐ 3	☐ 1 ☐ 2 ☐ 3
Fatigue	☐ 0 ☐ 1 ☐ 2 ☐ 3	☐ 1 ☐ 2 ☐ 3
Muscle or body aches	☐ 0 ☐ 1 ☐ 2 ☐ 3	☐ 1 ☐ 2 ☐ 3
Headache	☐ 0 ☐ 1 ☐ 2 ☐ 3	☐ 1 ☐ 2 ☐ 3
Sore Throat	☐ 0 ☐ 1 ☐ 2 ☐ 3	☐ 1 ☐ 2 ☐ 3
Congestion or runny nose	☐ 0 ☐ 1 ☐ 2 ☐ 3	☐ 1 ☐ 2 ☐ 3
Nausea	☐ 0 ☐ 1 ☐ 2 ☐ 3	☐ 1 ☐ 2 ☐ 3
Vomit (24 hours)	☐ 0 (no vomiting) ☐ 1 – 2 times ☐ 3 - 4 times ☐ 5 or more times	
Diarrhea (24 hours)	☐ 0 (no diarrhea) ☐ 1 – 2 times ☐ 3 - 4 times ☐ 5 or more times	
My sense of **taste** is	☐ same as usual ☐ less than usual ☐ I have no sense of taste	
My sense of **smell** is	☐ same as usual ☐ less than usual ☐ I have no sense of smell	
Comments:		
