# Supplementary material for: A composite symptoms severity score based on survey self-reports as a predictor of SARS-CoV-2 infection and viral load
Source: BMC Infect Dis. 2025 Sep 23;25:1116. doi: 10.1186/s12879-025-11653-4 (PMC12455794; doi:10.1186/s12879-025-11653-4)
Supplement: Supplementary file 2 — Supplementary Material 2. [file 12879_2025_11653_MOESM2_ESM.docx]

**Supplementary Material**

**A Composite Symptoms Severity Score based on Survey Self-Reports as a Predictor of SARS-CoV-2 Infection and Viral Load**

Damian Diaz^1^, Jesse A. Canchola^2^, Ana M. Groh^1^, Tuna Toptan^3^, Daniel Jarem^2^, Alison L. Kuchta^2^, Priscilla Moonsamy^2^, Annemarie Berger^3^*, Maria J. G. T. Vehreschild^1,4^, Sandra Ciesek^3,4^

^1^ Goethe University Frankfurt, University Hospital Frankfurt, Department of Internal Medicine, Infectious

Diseases, Frankfurt am Main, Germany

^2^ Roche Molecular Systems, Pleasanton, CA, USA

^3^ Institute of Medical Virology, Goethe University Frankfurt, University Hospital Frankfurt, Frankfurt am Main, Germany

^4^ Fraunhofer Institute for Translational Medicine and Pharmacology ITMP, Frankfurt am Main, Germany

**S.1. Symptoms Severity Score (SS3) Distribution**

To measure symptoms severity, a symptoms severity score (S3) was derived from the BRAVA self-reports following a standardized approach. The first step involved assigning values to the reported symptoms based on their severity. Patients with up to 12 reported symptoms per visit were included in the analysis. The severity values for the initial eight symptoms, including cough, breathing difficulty, fatigue, body aches (myalgia), headache, sore throat, congestion/runny nose, and nausea, were categorized as follows: "None"=0, "Mild"=1, "Moderate"=2, and "Severe"=3. For the next two possible symptoms, vomit and diarrhea, the assigned values were: "0"=0, "1-2"=1.5, and "3-4"=3.5. The last two potential symptoms, loss of taste and loss of smell, were classified as: "Same as usual"=0, "Less than usual"=1.5, and "No taste or smell"=3.5.

To obtain a comprehensive measure of symptoms severity, a symptoms severity score (S3) was calculated by summing the reported values across all visits for each subject. The resulting distribution of S3 scores exhibited a right-skewed pattern (**Figure S.1**).

**Figure S.1. Distribution of Symptoms Severity Score**


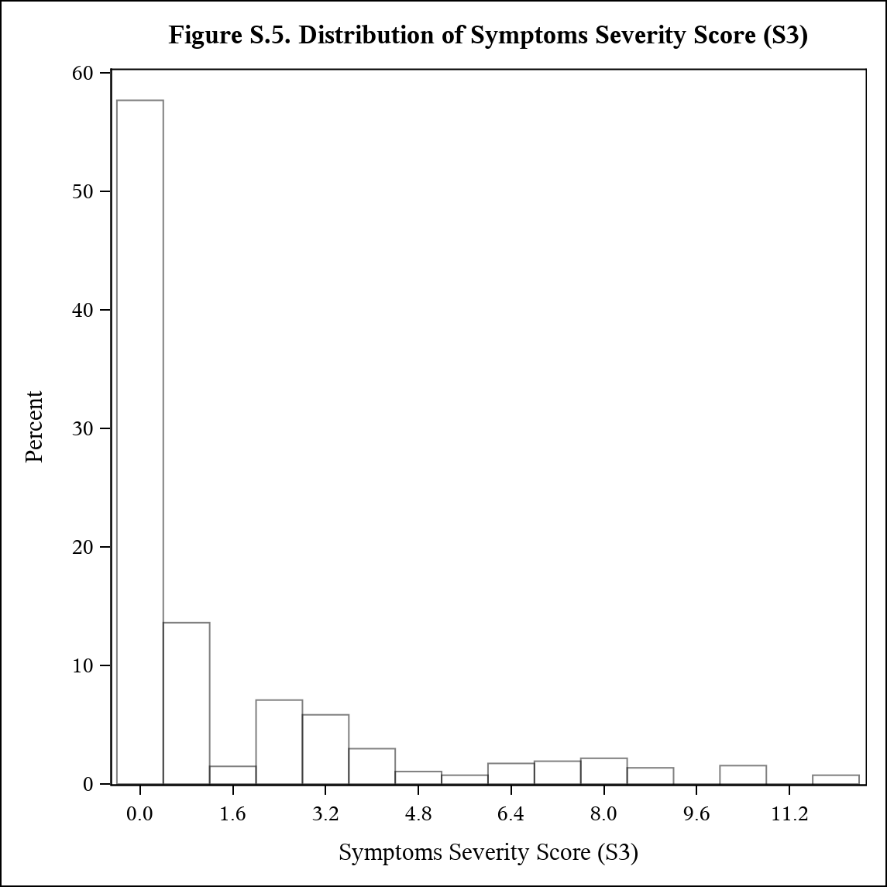


The internal consistency of the S3 construct, a measure of reliability, was assessed using Cronbach's alpha (Ca). The Ca value including all 12 symptoms was 0.7. The range of 0.70 to 0.79 suggests moderate internal consistency, indicating that the items in the scale construct are reasonably correlated with each other (1).

To confirm the underlying dimensionality of the reduced 12-item set, a factor analysis with varimax rotation was conducted. The results affirmed that the reduced set of symptoms represented a single underlying dimension defined here as the Symptoms Severity Score or S3.

To provide a general overview of symptoms severity, the S3 scores were further categorized into three groups: an S3 score of 0 indicated asymptomatic individuals, a score of 1-2 denoted mildly symptomatic individuals, and a score of 3 or higher represented severely symptomatic individuals.

**S.2. Reference Curve Calculation Results for Obtaining Quantitative VL Estimates**

To explore the VL dynamics of SARS-CoV-2 with respect to self-reported symptoms severity and number of symptoms for this study, the cycle threshold (Ct) values were used to obtain quantitative estimates of VL using reference curves. Three reference curves were developed as a robustness check whilst containing the PCR reaction efficiencies (Wilhelm & Pingoud, 2003) between 90% and 110% as calculated by formula (1):


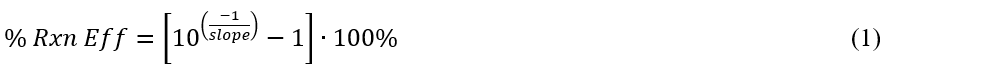


The first curve is the average of 7 standard curves after the analysis from Challenger et al. (2022; see their Supplementary Figure 1) upon reversing their x- and y-axes per standard reference curve analysis (mirroring the fact that Ct is inversely linearly related to log_10_ quantitation):

 Ct = 47 - 3.5 log_10_(Quant_JC_) (2)


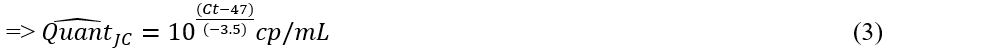


 which represents a PCR reaction efficiency of:


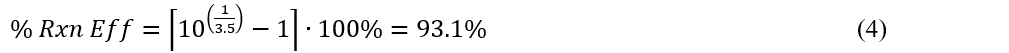


Contrast this with the earlier paper by Zou et al (2020) which found about 110% reaction efficiency PCR reaction:


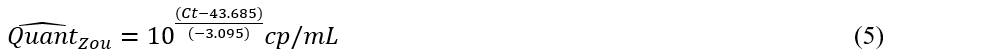


An additional quantitation variable with 100% reaction efficiency, viz., regression slope = -3.323 with the intercept halfway between those from Zou and Challenger, viz., (47+43.685)/2 = 45.3:


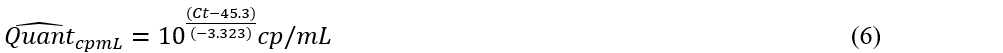


To ensure the robustness and reliability of our results, we chose the middle ground between the two published curves (100% efficiency with the average of the two intercepts) thereby enhancing the accuracy and confidence in the outcomes of our study.

 
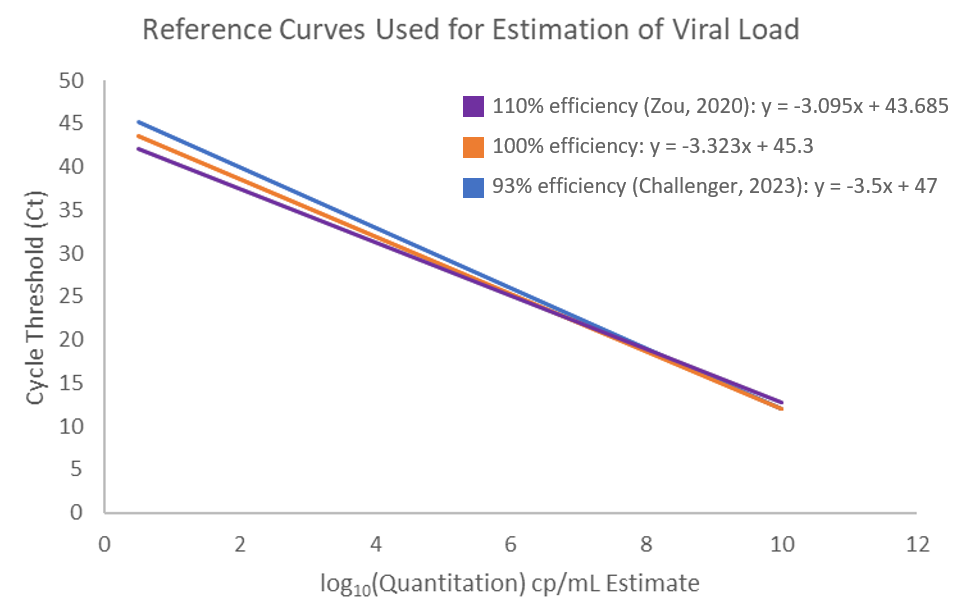


Reaction Efficiency is calculated as
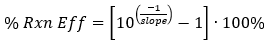
 where the Ct value is on the Y-axis and log_10_(quant) on the X-axis. Intercept for 100% Reaction Efficiency is calculated as the mean of those from previously published curves (2, 3).

**S.3. Continuous Outcome Results**

**S.3.1. Descriptive Statistics of log_10_ VL by S3C and NoSC**

The descriptive statistics of log_10_ VL by S3C and NoSC, adjusted for multiple visits within the patient are noted in Table 4. The mean VL increased monotonically from the lower categories (asymptomatic for S3C and 0 symptoms for NoSC) to the upper categories (severe symptoms for S3C to 3+ symptoms for NoSC).

**Table S.1.** Mean, SE, and 95% CI of log_10_ cp/mL E gene SARS-CoV-2 by **S3C** and **NoSC**, adjusted for multiple visits within the patient (n=89).

|  | **Symptoms Severity Category (all symptoms)** | **N** | **Mean  log_10_ cp/mL** | **Standard Error** | **95% CI for Mean** | |  |
| --- | --- | --- | --- | --- | --- | --- | --- |
|  |  |  |  |  |  |  |  |
| **S3C** | Asymptomatic | 30 | 4.48 | 0.32 | 3.74 | 5.21 |  |
|  | Mild symptoms | 26 | 4.52 | 0.22 | 4.01 | 5.04 |  |
|  | Severe symptoms | 33 | 5.31 | 0.34 | 4.51 | 6.10 |  |
|  |  |  |  |  |  |  |  |
| **NoSC** | 0 Symptoms | 30 | 4.48 | 0.32 | 3.74 | 5.21 |  |
|  | 1-2 Symptoms | 29 | 4.59 | 0.19 | 4.14 | 5.04 |  |
|  | 3+ Symptoms | 30 | 5.32 | 0.35 | 4.52 | 6.13 |  |

CI, confidence interval; cp/m, copies per milliliter; NoSC, Number of Symptoms Category; SE, standard error; S3C, three category symptoms severity score

**S.3.2. Model 3A for log_10_ VL with predictor S3Log**

Figure S.2 shows the Model 3A results of the continuous-level analyses for both the S3Log predictor and log_10_ VL outcome. Note that because the log_10_ transformation was performed on the symptoms severity score, the “0” symptoms severity score is undefined for this model. Nevertheless, the predicted estimates are monotonically increasing with symptoms severity score inputs.

**Figure S.2.** Results of GEE Model 3A (with compound symmetric correlation structure) for log_10_ cp/mL E gene SARS-CoV-2 outcome (**log_10_ VL**) with log_10_ Symptoms Severity Score (**S3Log**) as a predictor (n=89).

log_10_(SARS-CoV-2) = 4.6620 + 0.5172· log_10_(Symptoms Severity Score)

GEE, generalized estimating equation accounting for multiple visits within patient

**S.3.3. Model 4A for log_10_ VL with Predictor NoSLog**

Figure S.3 shows the results of the Model 4A continuous-level analyses for both the NoSLog predictor and log_10_ VL outcome. Note that because the log_10_ transformation was performed on the number of symptoms, the “0” symptoms input is undefined for this model. Nevertheless, the predicted log_10_ VL estimates are monotonically increasing with the number of symptoms inputs.

**Figure S.3.** Results of GEE Model 4A (with compound symmetric correlation structure) for log_10_ cp/mL E gene SARS-CoV-2 outcome (**log_10_ VL**) with log_10_ Number of Symptoms (**NoSLog**) as a predictor (n=89)

GEE, generalized estimating equation accounting for multiple visits within patient

**S.3.4. Model 3B for log_10_ VL with predictor S3**

Figure S.4 shows the results of the Model 3B continuous-level analyses for both the S3 predictor and log_10_ VL outcome. The log_10_ VL predicted estimates are monotonically increasing with symptoms severity score inputs.

**Figure S.4.** Results of GEE Model 3B (with compound symmetric correlation structure) for log_10_ cp/mL E gene SARS-CoV-2 outcome (**log_10_ VL**) with Symptoms Severity Score (**S3**) as a predictor (n=89).

log10(SARS-CoV-2) = 4.6425 + 0.0659 · (Symptoms Severity Score)

GEE, generalized estimating equation accounting for multiple visits within patient

**S.3.5. Model 4B for log_10_ VL with Predictor NoS**

Figure S.5 shows the results of the continuous-level analyses for both the NoS predictor and log_10_ SARS-CoV-2 VL outcome. The SARS-CoV-2 VL predicted estimates are monotonically increasing with the number of symptoms inputs.

**Figure S.5** Results of GEE Model 4B (with compound symmetric correlation structure) for log_10_ cp/mL E gene SARS-CoV-2 outcome (**log_10_ VL**) with Number of Symptoms (**NoS**) as a predictor (n=89).

GEE, generalized estimating equation accounting for multiple visits within patient

**References**

1. Nunnally J, Bernstein I. Psychometric theory 3rd ed: McGraw-Hill; 1994.

2. Challenger JD, Foo CY, Wu Y, Yan AWC, Marjaneh MM, Liew F, et al. Modelling upper respiratory viral load dynamics of SARS-CoV-2. BMC Medicine. 2022;20(1):25.

3. Zou L, Ruan F, Huang M, Liang L, Huang H, Hong Z, et al. SARS-CoV-2 Viral Load in Upper Respiratory Specimens of Infected Patients. The New England journal of medicine. 2020;382(12):1177-9.
